# Interpretable Machine Learning Model for Locoregional Relapse Prediction in Oropharyngeal Cancers

**DOI:** 10.3390/cancers13010057

**Published:** 2020-12-28

**Authors:** Paul Giraud, Philippe Giraud, Eliot Nicolas, Pierre Boisselier, Marc Alfonsi, Michel Rives, Etienne Bardet, Valentin Calugaru, Georges Noel, Enrique Chajon, Pascal Pommier, Magali Morelle, Lionel Perrier, Xavier Liem, Anita Burgun, Jean Emmanuel Bibault

**Affiliations:** 1 INSERM UMR 1138 Lab, Centre de Recherche des Cordeliers, Medical Informatics Unit, Georges Pompidou European Hospital—20 rue Leblanc, 75015 Paris, France; anita.burgun@aphp.fr (A.B.); jean-emmanuel.bibault@aphp.fr (J.E.B.); 2 Radiation Oncology Department, Georges Pompidou European Hospital, 20 rue Leblanc, 75015 Paris, France; philippe.giraud@aphp.fr (P.G.); eliot.nicolas@aphp.fr (E.N.); 3 Radiation Oncology Department, Montpellier Cancer Institute (ICM), 34090 Montpellier, France; pierre.boisselier@icm.unicancer.fr; 4 Radiation Oncology Department, Sainte Catherine Institute, 84918 Avignon, France; m.alfonsi@isc84.org; 5 Radiation Oncology Department, Claudius Regaud Institute, 31300 Toulouse, France; rives.michel@iuct-oncopole.fr; 6 Radiation Oncology Department, René Gauducheau Cancer Centre, 44800 Saint-Herblain, France; etienne.bardet@ico.unicancer.fr; 7 Radiation Oncology Department, Curie Institut, 75005 Paris, France; valentin.calugaru@curie.fr; 8 Institut de Cancerologie de Strasbourg, 17 rue Albert Calmette BP 23025, 67033 Strasbourg CEDEX, France; gnoel@strasbourg.unicancer.fr; 9 Radiation Oncology Department, Eugène Marquis Cancer Centre, 35000 Rennes, France; e.chajon@i-l-c.fr; 10 Radiation Oncology Department, Leon Bérard Cancer Centre, 69008 Lyon, France; pascal.pommier@lyon.unicancer.fr (P.P.); lionel.perrier@lyon.unicancer.fr (L.P.); 11 GATE L-SE UMR 5824, Lyon University, Léon Bérard Cancer Center, F-69008, Lyon, France; magali.morelle@lyon.unicancer.fr; 12 Radiation Oncology Department, Oscar Lambret Cancer Centre, 59000 Lille, France; x-liem@o-lambret.fr

**Keywords:** oropharyngeal cancer, head and neck, machine learning, XGBoost, radiomics

## Abstract

**Simple Summary:**

Machine learning may be used to personalize cancer care. However, physicians need interpretability to understand and use a predictive model powered by machine learning. We present a radiomics based model, interpretable for each patient, trained on an American multicentric cohort that yielded a 92% predictive value for relapse at 18 months in oropharyngeal cancers when tested on an external multicentric prospective French cohort.

**Abstract:**

Background: There is no evidence to support surgery or radiotherapy as the best treatment for resectable oropharyngeal cancers with a negative HPV status. Predictive algorithms may help to decide which strategy to choose, but they will only be accepted by caregivers and European authorities if they are interpretable. As a proof of concept, we developed a predictive and interpretable algorithm to predict locoregional relapse at 18 months for oropharyngeal cancers as a first step towards that goal. Methods: The model was based on clinical and Pyradiomics features extracted from the dosimetric CT scan. Intraclass correlation was used to filter out features dependant on delineation. Correlated redundant features were also removed. An XGBoost model was cross-validated and optimised on the HN1 cohort (79 patients), and performances were assessed on the ART ORL cohort (45 patients). The Shapley Values were used to provide an overall and local explanation of the model. Results: On the ART ORL cohort, the model trained on HN1 yielded a precision—or predictive positive value—of 0.92, a recall of 0.42, an area under the curve of the receiver operating characteristic of 0.68 and an accuracy of 0.64. The most contributory features were shape Voxel Volume, grey level size zone matrix Small Area Emphasis (glszmSAE), gldm Dependence Non Uniformity Normalized (gldmDNUN), Sex and Age. Conclusions: We developed an interpretable and generalizable model that could yield a good precision—positive predictive value—for relapse at 18 months on a different test cohort.

## 1. Introduction

Resectable oropharyngeal cancers can be treated either by definitive chemoradiotherapy or surgery [1]. There is currently no evidence to prefer one of the two options. In case of non-complete response after radiotherapy, salvage surgery can be performed at the cost of an extended decrease in quality of life due to chronic adverse events [2,3]. A predictive model for relapse after chemoradiotherapy in this setting may help to choose between both alternatives. However, acceptability of such a model is key for its implementation. For physician and the European Union, such a model should be interpretable to be acceptable since it would provide guidance for high responsibility decisions [4]. As a result, the black box effect of neural networks may be an obstacle to its acceptability for clinical prediction and decision support [5]. Radiomics features extraction provide data on various quantitative aspects of a region of interest (ROI). Aerts et al. and various other studies showed a link between radiomics features and genomic or histologic tumour characteristics such as HPV status or metastatic probability, with an added value to HPV and TNM for prognosis stratification [6,7,8,9,10,11]. A frequent drawback of these studies was their retrospective and frequently monocentric data, while radiomic features extraction may depend on the CT acquisition parameters (CT scan definition, contrast injection protocols, image reconstruction). As a result, this creates a risk of non-generalisability of such models. We thus created an interpretable predictive model of relapse at 18 months after chemoradiation for oropharyngeal cancers with an external validation on a French prospective and multicentric cohort.

## 2. Results

### 2.1. Population

**Figure 1:**
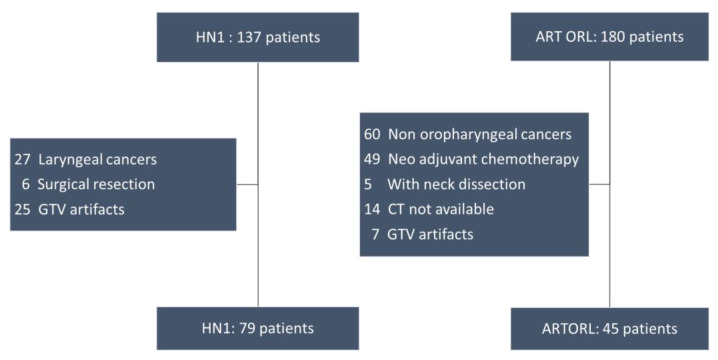
Flow charts of cohorts HN1 and ART ORL.

In HN1, 79 patients had an oropharyngeal cancer and were included in the training cohort. We faced patient attrition in the ART ORL cohort (Figure 1).

The ART ORL cohort initially included 180 patients, 120 of whom had an orophayngeal cancer. Among oropharyngeal cancers, 49 had a neoadjuvant treatment, 5 had a neck dissection and 21 had either a non-transmitted CT or artefacts on more than 50% of the Gross Tumour Volume (GTV). As a result, 45 patients were included in the test cohort. Moreover, HPV was not collected in ART ORL. Clinical characteristics such as age, American Joint Committee of Cancer (AJCC) and TNM stage and performance status were not statistically different between the two cohorts. HPV status, however, could not be compared (Appendix A).

**Figure 2:**
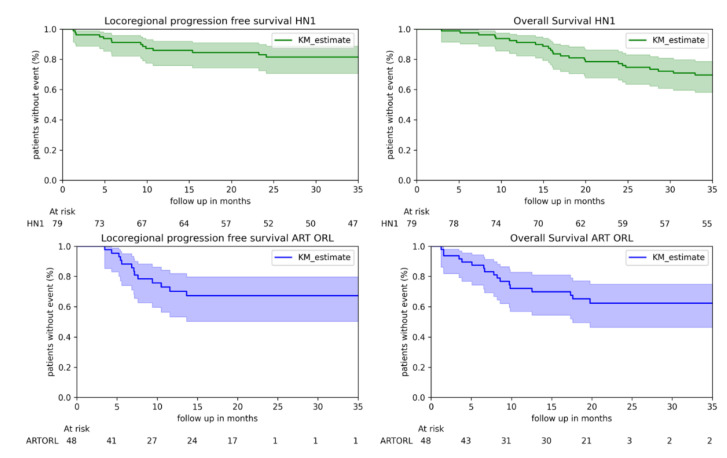
Kaplan Meier estimates, for HN1 and ART ORL cohorts.

Kaplan–Meier estimates of overall survival and locoregional progression-free survival are presented in Figure 2. The follow up in the ART ORL cohort stopped at 3 years, and it appears that the confidence intervals increased at the end of the follow up of ART ORL due to censored data.

### 2.2. Performance on ART ORL of a Model Trained on HN1

At 18 months, 20 patients of HN1 had a locoregional relapse (25%) vs. 26 (58%) of ART ORL.

**Figure 3:**
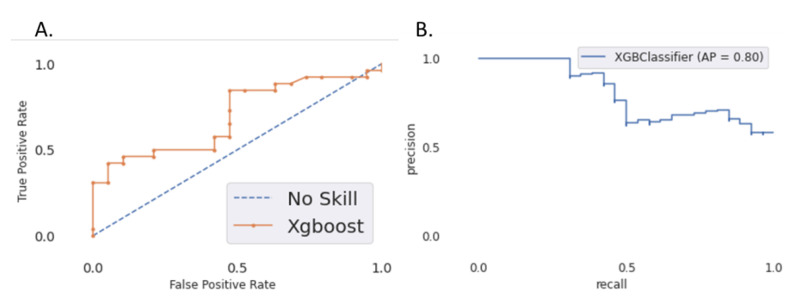
(**A**) ROC curve for the 18-month locoregional relapse prediction; (**B**) precision–recall curve on the test set (ART ORL).

The XGBoost model was trained using “mean average precision” as eval_metric and “precision” for optimization. The hyperparameters obtained after optimization are detailed in Appendix B. It yielded a precision (predictive positive value) of 0.92 in the test set with only one false positive (falsely classified as relapse). The recall (sensitivity) was 0.42, and the AUC of the ROC curve was 0.68. The AUC of the precision–recall curve was 0.79 (Figure 3). The accuracy was 0.64.

### 2.3. Interpretability with Shapley Values

**Figure 4:**
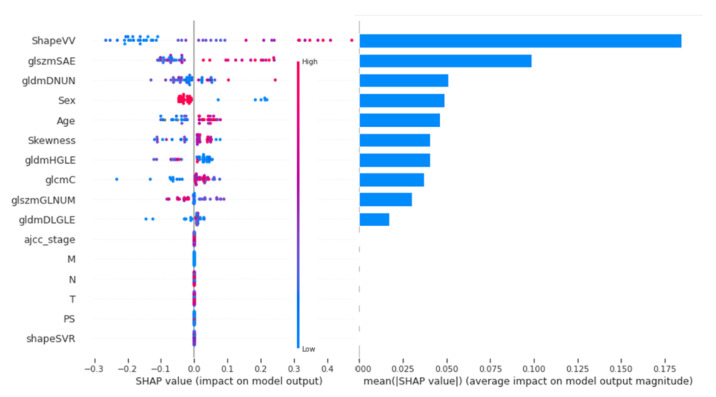
Ranking of features on their Shapley Value. gldmDNUN: gldm Dependence Non Uniformity Normalized; gldmDLGLE: gldm Large Dependence Low Grey Level Emphasis; glcmC: glcm Correlation; glszmGLNUN: glszm Grey Level Non Uniformity Normalized; glszmSAE: glszm Small Area Emphasis.

The contribution of each feature on the whole dataset is shown in Figure 4 by order of importance.

**Figure 5:**
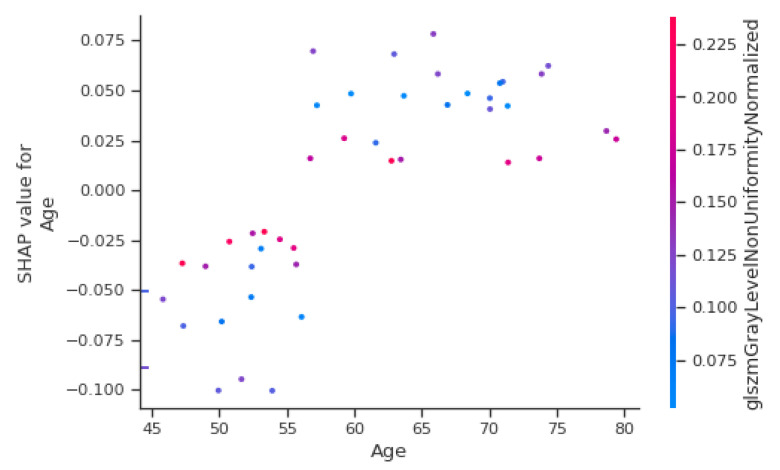
Shapley Value depending on age for each patient.

An explanatory table of most contributing radiomic features is in Appendix C. The most important clinical features are sex and age, in fourth and fifth position of importance behind three radiomics parameters. AJCC stage, T stage and N stage did not contribute to the model output. Figure 5 shows the Shapley Value of the age for each patient, with colour depending on the gldm Grey Level Non-Uniformity Normalized (gldmGLNUN), a radiomic feature increased in case of heterogeneity.

**Figure 6:**
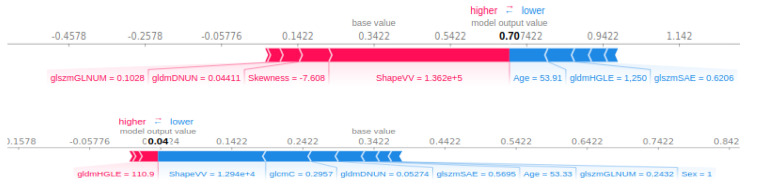
Contributions of features for a patient predicted as progression (higher) and a patient predicted without prediction (lower). gldmDNUN: gldm Dependence Non-Uniformity Normalized; gldmDLGLE: gldm Large Dependence Low Grey Level Emphasis; glcmC: glcm Correlation; glszmGLNUN: glszm Grey Level Non-Uniformity Normalized; glszmSAE: glszm Small Area Emphasis.

Below 57 years, the value is negative and thus appears to diminish the output probability, while above, the age contributes to the increase of the output. However, in the case of a high value of gldmGLNUN, the Shapley Value of age appears to tend towards zero. As a result, a high heterogeneity may take over the importance of age for the contribution to the output. All the features’ contribution to the output can be plotted for one single patient. For instance, in Figure 6, shape Voxel Volume (shapeVV) contributes to the increase in the predicted probability for progression, while a ten-times lower shapeVV contributes to a decrease in the predicted progression probability. In both cases, as seen previously, the age is below 57 and contributes to a diminished output probability.

## 3. Discussion

Our XGBoost model trained on HN1 yielded a 92% predictive value for progression of 18 months relapse for oropharyngeal cancers. The strength of this model is its training on an American multicentric cohort and a validation on a French multicentric prospective cohort, which gives hope for the generalizability of these performances. Furthermore, the intra class correlation (ICC) filtering of radiomic features and the multicentricity of cohorts make the remaining radiomic parameters robust. Due to this high positive predictive value, this model could be a first step towards a tool to help decision making and chooseing surgery for patients with a predicted relapse. However, the low recall and low accuracy make it obvious that a non-progression prediction is not to be taken into account.

However, several drawbacks may make this model unacceptable for a strategic clinical trial. First, HPV was not used as it was missing in the validation set. The AJCC staging used in the cohort was the 7th version, not yet taking HPV status into account. The number of patients was small compared to the number of features, with an important attrition in the validation cohort. Other known prognostic variables were missing, such as the histologic differentiation, the ulcerated or budding aspect of the lesion.

The second drawback is underlined thanks to the Shapley Values: the model the most contributive features are radiomic features, which are not easy to interpret. It was demonstrated that HPV status was correlated to tumour response and prognosis. It is difficult to assess the added value of radiomic features compared to HPV in our model. Yu et al. demonstrated that IBEX features SphericalDisproportion and MeanBreadth, correlated to sphericity and length, were associated with HPV: a simple and small volume had greater chances of being HPV-positive [9]. In our model, ShapeVV was correlated with volume and contributed most to the model, while clinical T, also correlated with size, was not. As a result, shapeVV may have a link with HPV status, which could explain its high contribution compared to the T stage. However, the shape volume ratio, close to IBEX’s SphericalDisproportion correlated with size, did not contribute to our model, which may impede this rationale.

Most other contributing radiomic features describe tumour heterogeneity, which may have a prognosis value [12]. Jong et al. [13] reported that the HPV status combined with a genomic profile was sufficient and better than clinical features to predict response to radiotherapy. As a result, heterogeneity features may be linked to this tumour profile. However, this remains to be proven.

We chose XGBoost since it has usually yielded the best performances on tabular data while being interpretable. Bryce et al. developed a deep learning model to predict 2y survival for head and neck cancers [14]. It yielded an AUC of the ROC curve of 0.78, precision and recall were not published and local interpretability was not possible. Ou et al. [12] used radiomics with a logistic regression model to yield an AUC of 0.67 without HPV status—comparable to our study—which increased to 0.78 when using HPV status. Zhang et al. [8] developed a regression model to predict 5y overall survival for nasopharyngeal cancer and yielded a C-Index of 0.776 using radiomics feature. Parmar et al. [15] also trained on HN1 and validated on HN2 cohort a Bayesian model (AUC: 0.67), a random forest classifier (AUC: 0.61) and a neural network (AUC: 0.62) based on radiomics with comparable AUC on with the same training cohort, with comparable AUC, but no published precision/recall, while these metrics are important in binary classification. Karadaghy et al. [16] used a decision forest architecture conducted on 33,065 patients to predict 5y overall survival. The AUC was 0.8, the precision was 71% and recall was 0.68.

To our knowledge, this is the first study to report a predictive model interpretable for each patient’s classification. Local interpretability is a key for predictive algorithms in clinical practice since they may provide support to decisions with a high responsibility burden and impact on a patient’s life. We computed the Shapley Value through its implementation in the shap package because this interpretation technique relied on a strong mathematical rationale to provide a feature contribution to the output and not to the loss function reduction, which is more clinically relevant. It provided the local and global explanation needed in the context of validation of the overall model and daily use for a single patient. shap provided a better understanding behind the model’s performance and revealed that it rests on radiomics features, which are not interpretable themselves as there is not enough evidence for a strong link between the most contributing radiomic features and a biologic parameter interpretable by clinicians. As a result, the black box effect may have moved from the model towards the features on which it is based, which may impede interpretability for clinicians and thus acceptability.

## 4. Materials and Methods

### 4.1. Population

We collected patients from the multicentric retrospective public American database of HN1 previously used by Aerts et al. [17,18] for training, available on The Cancer Imaging Archive and the French prospective multicentric cohort ART ORL of Bibault et al. [19] for validation. Kaplan–Meier estimates were performed to describe the two cohorts. Locoregional relapse was defined as a tumoral bed or cervical lymph node relapse. The locoregional survival was defined as the time between the 1st session of radiation therapy and the locoregional relapse or the end/loss of follow up. Patients had to be diagnosed with an oropharyngeal cancer treated with definitive chemoradiation without any neoadjuvant treatment nor systematic node dissection after chemoradiation. For radiomic features extraction, CT scans quality criteria were set: primitive tumours had to be visible on the dosimetric CT scan; if artefacts, they had to affect less than 50% of slices of the Gross Tumour Volume (GTV) according to guidelines by Ger et al. [20].

### 4.2. Radiomics Features

**Figure 7:**
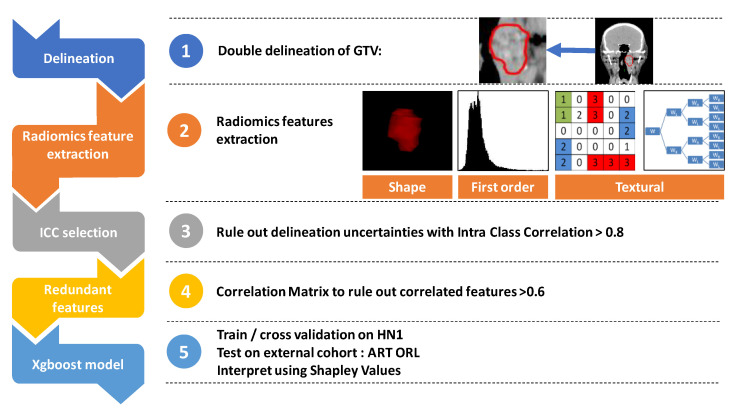
The study workflow.

Two contours of the GTV were made, each by a radiation oncologist blinded from the other’s delineation. Although acquisition parameters are known to influence radiomic features, metadata on acquisition parameters of HN1 were not available, so we could not assess the training cohort CT acquisition variability [21]. However, in the validation cohort (ART ORL) we had a homogenous tube voltage and section thickness. The X-ray tube current was variable (from 40 mA to 445 mA), as was the kernel convolution used (Appendix D). Spatial resampling was not performed prior to extraction. Radiomic features were extracted in a 3D fashion from each contours using Pyradiomics [7]. GTV ROI used for extraction were of sufficient size, ranging from 1.9 cm^3^ to 234 cm^3^ for HN1 and from 2.3 cm^3^ to 136.2 cm^3^ for ART ORL (Appendix E). Radiomic feature filtering was performed on the training cohort (HN1). First, only reproducible features between contours were kept to rule out features depending on delineation uncertainties as described by several authors [22,23,24]. Intraclass correlation above 0.8 was required for features between each contour. A correlation matrix of the remaining 42 radiomics features was used to avoid feature redundancies (Appendix F). If two features had a correlation of more than 0.6, one of them was removed. Figure 7 shows the workflow.

### 4.3. XGBoost

Since we handled tabular data with a need for interpretability, we used XGBoost (eXtreme gradient boosted tree) [25,26]. This classifier yielded one of the best performances in Kaggle classification competitions. It uses a combination of weak learners (decision trees). At each learning iteration, each new tree is integrated with the ensemble of weak learners depending on its contribution to the loss function reduction. It is in essence interpretable and thus fits our goal.

We used HN1 as the training cohort with a 5-fold cross-validation. A Bayesian optimization was performed (hyper parameters bound in Appendix G). The XGBoost objective was “binary: logistic”. The trained and optimized classifier was then tested on the ART ORL cohort, and we reported its performance on this test cohort. As it is a binary classifier, the most relevant parameters to report were precision and recall. The area under the curve (AUC) of the receiver operator characteristic (ROC) and the accuracy were also reported.

### 4.4. Shapley Value

The Shapley Value comes from game theory [27,28,29]. It is the assessment of the contribution of each variable of the model to its output (or prediction in our case). For a variable of interest, the output of each possible combination of other variables is collected. The difference between the average of all those possible outputs without the variable of interest and the model’s output when including the variable of interest is the Shapley Value of the variable. This allows one to quantify the impact of each variable on the prediction not only on a global level (on the overall population) but also locally (on a subset or one patient). Thus, it does not provide the importance of each feature to the loss function decrease but to the prediction result. Shapley Values for each variable are thus additive, which makes the contribution of each variable convertible to a share of the output classification probability. This provides an intuitive visualization for clinicians if this model should ever be used. The shap package developed by Lundberg [30] was used with TreeExplainer to compute Shapley Values and provide global and local explanatory visualization.

### 4.5. Ethical Committee

We reused data from the ART ORL prospective cohort. Patients were included in 2011, and the initial patient’s information did not encompass data reuse. As a result, we sent an information letter to patients stating their right to oppose the reuse of their data. The Ethical Board then granted access and we declared to the Commission Nationale d’Informatique et Liberté (CNIL—French data regulator) that our data processing complied with the reference method MR-004 of the CNIL. The study is registered in the national institute of health data (INDS, n° 5313160620).

## 5. Conclusions

Training an XGBoost model on HN1 and testing on ART ORL yielded a good positive predictive value for locoregional progression at 18 months. The small size of cohorts, due to attrition for ART ORL, and the reduced contribution of clinical interpretable variables may impede the acceptability of this model for a clinical strategy trial. However, XGBoost and the Shapley value provide a robust way to build interpretable classifiers against the backdrop of a requirement of interpretability by clinicians and regulators due to the impact such models may have on clinical decision. Such interpretability requirements may spread towards the features contributing to prediction, which may also have to be interpretable or strongly linked to an interpretable concept.

## Figures and Tables

**Figure A1:**
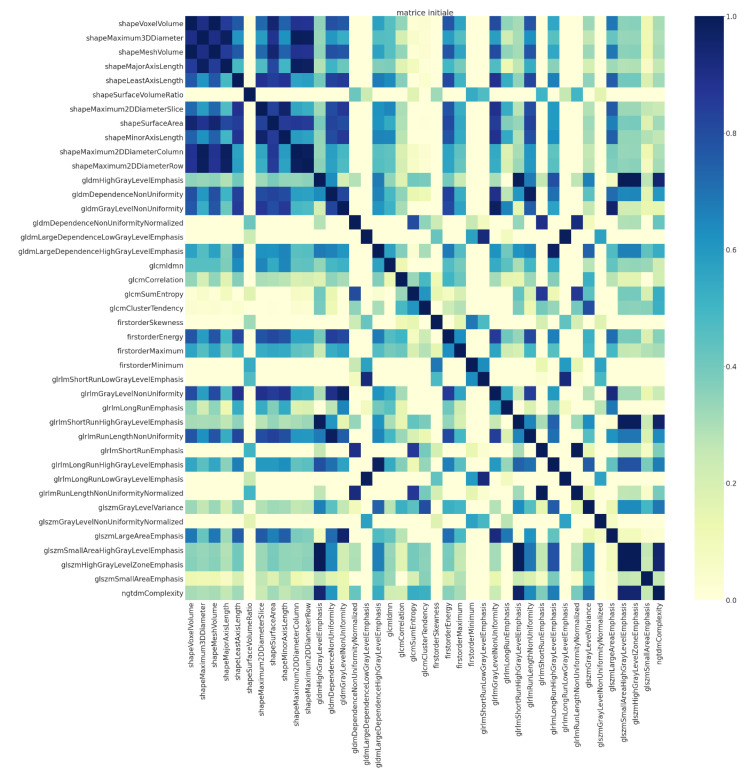
Correlation matrix for redundancy filtering among features.

## Data Availability

Research data are stored in an institutional repository and will be shared upon request to the corresponding author.

## Appendix A

**Table A1:** Patients’ characteristics.

Characteristics	Values	HN1 n (%)	ARTORL n (%)	*p*
**Sex**	**Male**	59 (75)	39 (87)	0.17
	**Female**	20 (25)	6 (13)	
**PS**	**0**	36 (46)	30 (67)	0.29
	**1**	33 (41)	14 (31)	
	**2**	2 (3)	0	
	**3**	1 (1)	0	
	**NC**	7 (9)	1 (2)	
**HPV**	**0**	50 (63)	0	non assessable
	**1**	22 (28)	0	
	**NC**	7 (9)	45 (100)	
**T**	**1**	10 (13)	4 (10)	0.44
	**2**	26 (33)	11 (24)	
	**3**	12 (15)	19 (42)	
	**4**	31 (29)	11 (24)	
**N**	**0**	21 (27)	17 (38)	0.44
	**1**	12 (15)	5 (11)	
	**2**	44 (56)	23 (51)	
	**3**	2 (2)	0	
**AJCC stage**	**I**	4 (5)	3 (7)	0.49
	**II**	8 (10)	4 (9)	
	**III**	12 (15)	11 (24)	
	**IVa**	48 (61)	27 (60)	
	**IVb**	7 (9)	0	

Bold: separate index of lines from values.

## Appendix B

**Table A2:** Radiomics hyperparameters after Bayesian optimization.

Hyper Parameter	Value
colsample_bytree	0.740802
gamma	1.050870
learning_rate	0.755228
max_depth	4
min_child_weight	3.342836
n_estimators	807
reg_alpha	0.145084
reg_lambda	0.287761
scale_pos_weight	6.955609
subsample	0.924808

## Appendix C

**Table A3:** Explanatory table of contributive radiomics features.

Radiomic Feature	Formula	Explanation
**GLDM: Grey level Dependance Matrix:** Dependencies between a central voxel and voxels at a given length. Dependency is defined by a maximal spread of density between those voxels. *N_g_* number of grey levels *N_d_* number of discreet lengths in the image *N_z_* number of dependencies zones in the image **P**(*i*,*j*) is the dependency matrix		
**gldmDNUN**	$\frac{\sum_{i=1}^{N_{g}}{\left(\sum_{j=1}^{N_{d}}P\left(i,j\right)\right)}^{2}}{N_{z}}$	Measures the grey level similarity in the ROI
**gldmDLGLE**	$\frac{\sum_{i=1}^{N_{g}}\sum_{j=1}^{N_{d}}\frac{P\left(i,j\right)j^{2}}{i^{2}}}{N_{z}}$	Distribution of large matrix of dependencies of low grey levels
**GLSZM: Grey Level Size Zone Matrix:** a grey level zone is a zone of contiguous voxels with a similar grey level. *N_g_* number of grey levels *N_s_* number of possible sizes of grey levels in the ROI *N_p_* number of voxel in the ROI *N_z_* numbe of zones in the image **P**(*i*,*j*) size of the zone (matrix)		
**glszmGLNUN**	$\frac{\sum_{i=1}^{N_{g}}{\left(\sum_{j=1}^{N_{s}}P\left(i,j\right)\right)}^{2}}{N_{z}^{2}}$	Variability of the grey levels of zones. A small value is in favour of image homogeneity
**glszmSAE**	$\frac{\sum_{i=1}^{N_{g}}\sum_{j=1}^{N_{s}}\frac{P\left(i,j\right)}{j^{2}}}{N_{z}}$	Number of small size zones
**Shape features**		
**ShapeVV**	$V_{voxel}=\sum_{k=1}^{N_{v}}V_{k}$	Sum of the volume of voxels of ROI
**shapeSVR**	$\frac{A}{V}$	ROI’s surface on its volume. A small ratio is linked to a simple shape such as a sphere

gldmDNUN: gldm Dependence Non Uniformity Normalized; gldmDLGLE: gldm Large Dependence Low Grey Level Emphasis; glszmGLNUN: glszm Grey Level Non Uniformity Normalized; glszmSAE: glszm Small Area Emphasis; shapeSVR: shape Surface Volume Ratio; ROI: Region of Interest. Bold: highlight labels of lines.

## Appendix D

**Table A4:** Acquisition parameters of ART ORL.

Parameters	ART ORL Acquisition Parameters	ICC > 0.9 in Berenguer et al. [21]
Kernel convolution	STANDARD: 23	41.2%
	FC17: 7	
	FC04: 4	
	FC03: 2	
	B30s: 1	
Section thickness	min: 2.5 mm	94.9%
	max:3 mm	
FOV	data not provided	75.1%
Milliamperage	min: 40 mA	78.5%
	max: 445 mA	
Tube voltage	min: 120	41.8%
	max: 135	

The most critical parameters had a low ICC when this parameter was modified by Berenguer et al.. While we had a high variation in milliamperage, this may not have a great impact on radiomic features extraction, while our variability of convolution kernel may have an impact since a variability did impact the ICC when measured by Berenguer et al. FOV Field of View, ICC: intra class correlation.

## Appendix E

**Table A5:** Descriptive data on GTV volumes.

	HN1 (mm^3^)	ARTORL (mm^3^)
**mean**	32,768	30,590
**std. dev.**	37,778	27,556
**minimum**	1896	2348
**25%**	9706	11,954
**50%**	20,613	20,117
**75%**	39,361	41,050
**maximum**	234,527	136,175

Bold: highlight labels of lines.

## Appendix G

**Table A6:** Hyperparamter bounds for Bayesian optimization.

XGBoost Hyper Parameters	Lower Bound	Upper Bound
**scale_pos_weight**	Y = 0/Y = 1	2 + Y = 0/Y = 1
**n_estimators**	10	1000
**learning_rate**	0.1	1
**min_child_weight**	1	10
**max_depth**	3	12
**subsample**	0	1
**colsample_bytree**	0.3	1
**gamma**	0	5
**reg_alpha**	1 × 10^−5^	0.75
**reg_lambda**	1 × 10^−5^	0.45

Y = 0: patient without relapse at 18 months, Y = 1 patients with relapse at 18 months. Bold: highlight labels of lines.
